# Physical activity accumulation along the intensity spectrum differs between children and adults

**DOI:** 10.1007/s00421-021-04731-3

**Published:** 2021-06-05

**Authors:** Timo Rantalainen, Nicola D. Ridgers, Ying Gao, Daniel L. Belavý, Eero A. Haapala, Taija Finni

**Affiliations:** 1 Faculty of Sport and Health Sciences and Gerontology Research Center, University of Jyväskylä, P.O. Box 35 (viv 289), 40014 Jyvaskyla, Finland; 2 Institute for Physical Activity and Nutrition (IPAN), School of Exercise and Nutrition Sciences, Deakin University, Geelong, Australia; 3 Department of Sports Science, College of Education, Zhejiang University, Hangzhou, China; 4 Faculty of Sport and Health Sciences, Neuromuscular Research Center, University of Jyväskylä, Jyväskylä, Finland; 5 Institute of Biomedicine, School of Medicine, University of Eastern Finland, Kuopio, Finland; 6 Hochschule für Gesundheit (University of Applied Sciences), Division of Physiotherapy, Gesundheitscampus 6-8, 44801 Bochum, Germany

**Keywords:** Wearable, Activity, Actigraphy, Mean amplitude deviation

## Abstract

**Purpose:**

Detailed exploration of physical activity accumulation with fine grading along the intensity spectrum has indicated the potential pragmatic utility of such an approach. However, it is currently unclear what sorts of accumulation patterns along particular intensity bands are found in the children and adult populations. Therefore, we conducted a comparison of activity accumulation in specific intensity bands between four distinct populations: children, adults with sedentary lifestyles, habitual joggers, habitual marathon runners.

**Methods:**

Free-living waist-worn accelerometry records from 28 children aged 7 to 11, and 61 adults aged 25 to 35 were analysed. Activity intensity was evaluated in 5 s non-overlapping epochs as mean amplitude deviation (MAD) and normalised to acceleration intensities corresponding to walking at 3 metabolic equivalents of a task (METs). Adult data were normalised to 0.091 g MAD based on literature, and data from children to 0.170 g MAD based on laboratory experimentation. The normalised epoch values were divided into 100 intensity gradations.

**Results:**

Children accumulated more activity in 0.74 to 1.58 normalised acceleration intensities (all *p* < 0.005) compared to adults. Adult joggers/runners accumulated more activity in normalised acceleration intensities from 7.1 to 11.1 compared to the other groups (*p* < 0.008).

**Conclusion:**

The primary bulk of children’s free-living activities are of relatively low intensity not likely to provoke cardiometabolic improvement. These sorts of explorations could be used in informing intervention development aiming at optimising healthy development. Evidence is mounting to justify randomised controlled trials based on intervention targets identified based on exploring the intensity spectrum.

**Supplementary Information:**

The online version contains supplementary material available at 10.1007/s00421-021-04731-3.

## Introduction

Contemporary accelerometer-assessed physical activity analysis is based on summarising the day in non-overlapping consecutive one second to 1-min intervals (epochs), and typically categorising these epochs into one of four categories; sedentary behaviour, light activity, moderate activity, and vigorous activity (Sievänen and Kujala 2017; Migueles et al. 2021). Moderate and vigorous activity minutes are subsequently pooled to provide an indicator of total physical activity-related energy expenditure (Plasqui et al. 2013). However, considering the day in 1-min epochs may be problematic when activity engagement is sporadic (e.g. children’s activity patterns Bailey et al. 1995; Aadland et al. 2020)). Moreover, total activity-related energy expenditure is not the only physiological stimulus driving homeostasis, and the categorisation into only four categories may not have sufficient intensity resolution for other physiological targets.

For example, the known physiological underpinnings of skeletal adaptation to mechanical loading (Ahola et al. 2010; Jämsä et al. 2011) indicate that only a few high-impact loading cycles (e.g. 50 jumps) a few times (e.g. thrice) per week are required to elicit a positive bone adaptation (Niu et al. 2010). To capture this physiological stimulus using accelerometers, Ahola and colleagues developed an analytical approach, which, instead of targeting energy expenditure estimates, rather identified impact peaks with 31 intensity gradations, and summarised the peaks into a daily impact score (Ahola et al. 2010). Aadland et al. (2018b) and we have extended this fine gradation of intensity spectrum approach into physical activity analyses to explore whether specific parts of the intensity band are positively associated with particular health indicators (Belavy et al. 2018; Aadland et al. 2018b, 2020; Savikangas et al. 2020). We reported that vertebral marrow fat fraction was positively associated with intensities corresponding to walking and jogging but negatively associated with intensities corresponding to sedentary activities in adults (Belavy et al. 2018). Similarly, Aadland and colleagues showed that a particular intensity band (5000 to 7000 counts/minute) was more strongly associated with cardiometabolic health indicators among children than the other intensity bands (Aadland et al. 2018b). Rowlands and colleagues have developed a method similarly based on fine gradation of the intensity spectrum (Rowlands et al. 2018), and demonstrated that the accumulation pattern of the intensity histogram contains meaningful information related to health indicators in children (Rowlands et al. 2019, 2020). However, instead of probing particular intensity bands, they represented the overall accumulation pattern by the slope of the histogram (Rowlands et al. 2018). While some progress has been made in this space e.g. by Aadland and colleagues (e.g. Aadland et al. 2018b, 2020), and Rowlands and colleagues (e.g. Rowlands et al. 2018, 2020), there is still a relative dearth of exploration of the finely graded activity intensity spectrum in the literature to date for children.

Children have been observed to be sporadic in their physical behaviour (Bailey et al. 1995), and accumulate more total daily physical activity than adults (Troiano et al. 2008). However, due to categorising the day into just four classes of behaviour the extant literature does not offer a well-rounded understanding of activity accumulation in specific intensity bands and consequently does not provide an opportunity to identify potential intervention targets for outcomes other than total energy expenditure. Moreover, it may be that due to the sporadic nature of children’s physical behaviours, the use of a 1-min epoch may average out some of the short high-intensity bursts (Aadland et al. 2020) but it is unclear whether this is the case more so in children than in adults. Therefore, the purpose of the present paper was to explore the intensity spectrum in four distinct populations; adults with sedentary lifestyles, habitual joggers, habitual marathon runners, and children. It was hypothesised that the joggers and runners would accumulate activity within a relatively narrow intensity band corresponding to their jogging/running and that the children would be more active compared to adults in general. We also explored two alternate epoch lengths (5 s and 1 min) to evaluate any potential epoch length effects between children and adults. These explorations would be expected to contribute in identifying potential intensity range intervention targets to optimise children’s health and development.

## Methods

Data were drawn from two projects; the Children’s Physical Activity Spectrum (CHIPASE) study and the Spine Activity Study (SAP). Details of these studies have been reported elsewhere (Belavý et al. 2017; Belavy et al. 2018, 2019; Rantalainen et al. 2018; Gao et al. 2019). Briefly, thirty-five children from Jyväskylä (Finland) region aged 7 to 11 years of age assented, and returned informed written parental consent to participate in CHIPASE, and seventy-nine 25 to 35-year-old men and women from the Melbourne metropolitan area (Victoria, Australia) provided written informed consent to participate in SAP. Both trials included convenience samples recruited by posting advertisements to appropriate channels (social media, bulletin boards) and through word of mouth. Participants who provided at least one day of at least 8 h wear-time of accelerometry were included, resulting in 28 children and 61 adults being included in the analyses in the present paper. Participants in both studies were required to be in good general health (Belavý et al. 2017; Gao et al. 2019), and in the SAP study three specific cohorts were targeted; no sports group who self-reported less than 150 min of moderate-to-vigorous physical activity per week, joggers who ran 20 to 40 km per week, and marathon runners, who ran at least 50 km per week (Belavý et al. 2017). Both studies were approved by the appropriate ethical review boards (CHIPASE: Ethics Committee of the University of Jyväskylä; SAP: Deakin University Human Ethics Advisory Group—Health). Both studies were conducted in agreement with the Helsinki declaration.

Both studies included prolonged free-living accelerometry monitoring with an accelerometer worn on the waist on an elastic belt for a minimum of five (CHIPASE) to seven (SAP) consecutive days. The accelerometer was worn during waking hours and removed for water-based activities. The CHIPASE study utilised the X6-1a (Gulf Coast Data Concepts Inc., Waveland, USA) device, and the SAP study utilised the GT3X + (ActiGraph LLC, Pensacola, FL, USA) device. Both devices sampled 3-dimensional accelerations at ≥ 40 samples per second, had a range of at least ± 6 times the acceleration caused by gravity (g) with at least 12-bit analog-to-digital discretisation, and have been shown to produce comparable results (Vähä-Ypyä et al. 2015a). Height and weight were measured using a stadiometer and an electronic scale in both studies.

The numerical accelerometry analysis pipeline comprised four steps (Appendix 1): (1) autocalibration (van Hees et al. 2014), (2) pre-processing the calibrated accelerations into 5 s non-overlapping mean amplitude deviation (MAD) based on resultant acceleration (Vähä-Ypyä et al. 2015a), which were subsequently also summarised minute by minute, (3) identification of non-wear, and (4) calculating a hundred bin daily histogram based on the 5 s and 1-min values when the accelerometer was worn. The means of the histograms from all recorded days per participant are reported as the result for both the 5 s epoch results and the 1-min epoch results.

Daily histograms normalised to the MAD intensity corresponding to walking at three METs were produced. The normalisation was undertaken to enable comparing activity accumulation along the intensity gradation between children and adults and should not be confused with actual MET values. That is, MAD intensities do not increase linearly with increasing metabolic load, and therefore the MAD intensity corresponding to walking at 3 METs actually has 3 MET energy demand but this cannot reasonably be extrapolated to 0 METs or to 6 METs. For example, sitting and standing still both result in essentially 0 MAD but produce 1 and 1.5 MET, respectively. Similarly, there is a discontinuity in transitioning from walking to running in MAD values whereas energy consumption does not exhibit such a marked (if any) discontinuity with the transition (Vähä-Ypyä et al. 2015a, b; Belavý et al. 2017). The caveat aside, the 0.091 g value reported by Vähä-Ypyä et al. (2015a) was used for normalisation for adults. For children, a value of 0.170 g, which corresponds to walking on a treadmill at 4 km/h and resulted in oxygen consumption of 3 times the measured resting oxygen uptake (Gao et al. 2019), was used. The daily 5 s epoch normalised values were divided into a 100 bin logarithmically equidistant histogram bins ranging from 0 to 15 as was done with the MAD values above except for replacing 2 with 15 as appropriate. The mean of the normalised daily histograms is reported as the outcome.

Means and standard deviations (SD) are presented. The chosen sample size enables detecting medium to large statistical effect size, which was considered reasonable for this explorative study. Rank-based non-parametric methods for analysing longitudinal data in factorial experiments (nparLD) from the nparLD R-package (Noguchi et al. 2012) were used to evaluate group (4 between participants [whole-plot] categories; children, no sport, joggers, runners), epoch length (2 between participants categories; 5 s, 1 min), and intensity bin (100 within-participant [sub-plot] categories; the histogram bin counts described above) main effects, and interactions. Specific groups were compared to each other in post-hoc testing by selecting the specific groups and re-running the nparLD again. Group differences for variables with no within-subject repeats (e.g. height, weight), and post-hoc tests to identify specific within-subject repeat differences between groups were evaluated with a Kruskal–Wallis test. When appropriate, Dunn tests were subsequently used to identify between groups post hoc differences. All p-values from all of the Kruskal–Wallis tests were pooled and adjusted due to multiple comparisons using the false discovery rate approach (Benjamini and Hochberg 1995). The significance level was set at *p* < 0.05. R (64-bit version 3.6.3, https://www.r-project.org/) was used for statistical analyses.

## Results

No statistically significant differences were observed in age (group means: 29.6 to 30.5 years-of-age), height (170 to 175 cm), or body mass (62.8 to 76.1 kg) between the adult groups (*p* > 0.05; Table 1). The mean age, height and body mass for the children were 9.3 years-of-age, 138 cm, and 33.1 kg, respectively. Daily accelerometer wear duration did not differ between groups (11.9 to 12.8 h/day, *p* = 0.131), but, in accordance with the study protocols, children had fewer days included in the analysis than adults (4.2 days versus at least 6 days for all adult groups, p < 0.05; Table 1).

**Table 1:** Descriptive characteristics, and accelerometer wear statistics

	Children	No sport	Joggers	Marathon runners
*N*	28	18	23	20
Age (years)	9.3 (1.4)	30.3 (3.5)	30.5 (3.2)	29.6 (3.8)
Height (cm)	138 (9)	174 (8)	175 (10)	170 (9)
Body mass (kg)	33.1 (7.1)	76.1 (17.9)	68.7 (11.2)	62.8 (10.3)
Wear duration (h/day)	11.9 (1.2)	12.1 (1.7)	12.6 (1.4)	12.8 (1.2)
Days included (days)	4.18 (2.23)	6.28 (1.96)	6 (2.26)	6.35 (1.5)

**Fig. 1:**
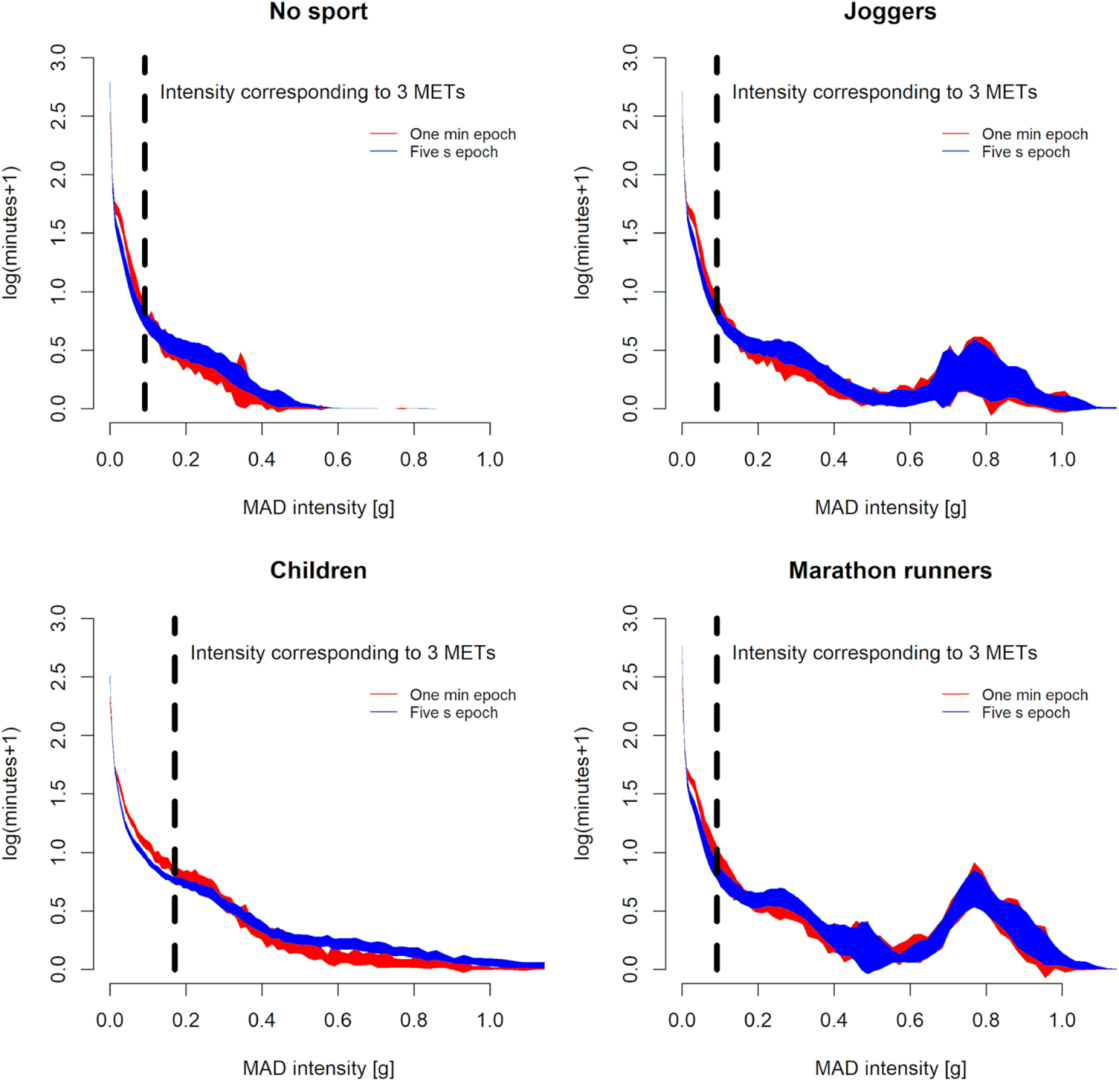
Visualisation of the activity intensity histograms based on 1 min non-overlapping activity intensity processing (red shading), and 5 s non-overlapping activity intensity mean amplitude deviation (MAD) processing (blue shading). The shaded areas correspond to the 95% confidence interval

Comparison of the effect of epoch length on accumulation pattern with nparLD indicated that all evaluated interactions were statistically significant (ATS 5.8 to 24.9; *df* = 3 to 35; *p* < 0.001). A statistically significant group, epoch length, and intensity bin main effect (ANOVA-type statistic [ATS] ranged from 34.8 to 832; *df* = 1 to 87; all *p* < 0.001) was also observed. Post hoc comparisons between epoch lengths within groups indicated statistically significant epoch length and intensity bin main effects (ATS 23.5 to 548; *df* = 1 to 7; all *p* < 0.001) and epoch length x intensity interaction (ATS 4.0 to 10.5, *df *= 9 to 11 among adult groups, 19.8, *df* = 11 for children; all *p* < 0.001) (Fig. 1).

**Fig. 2:**
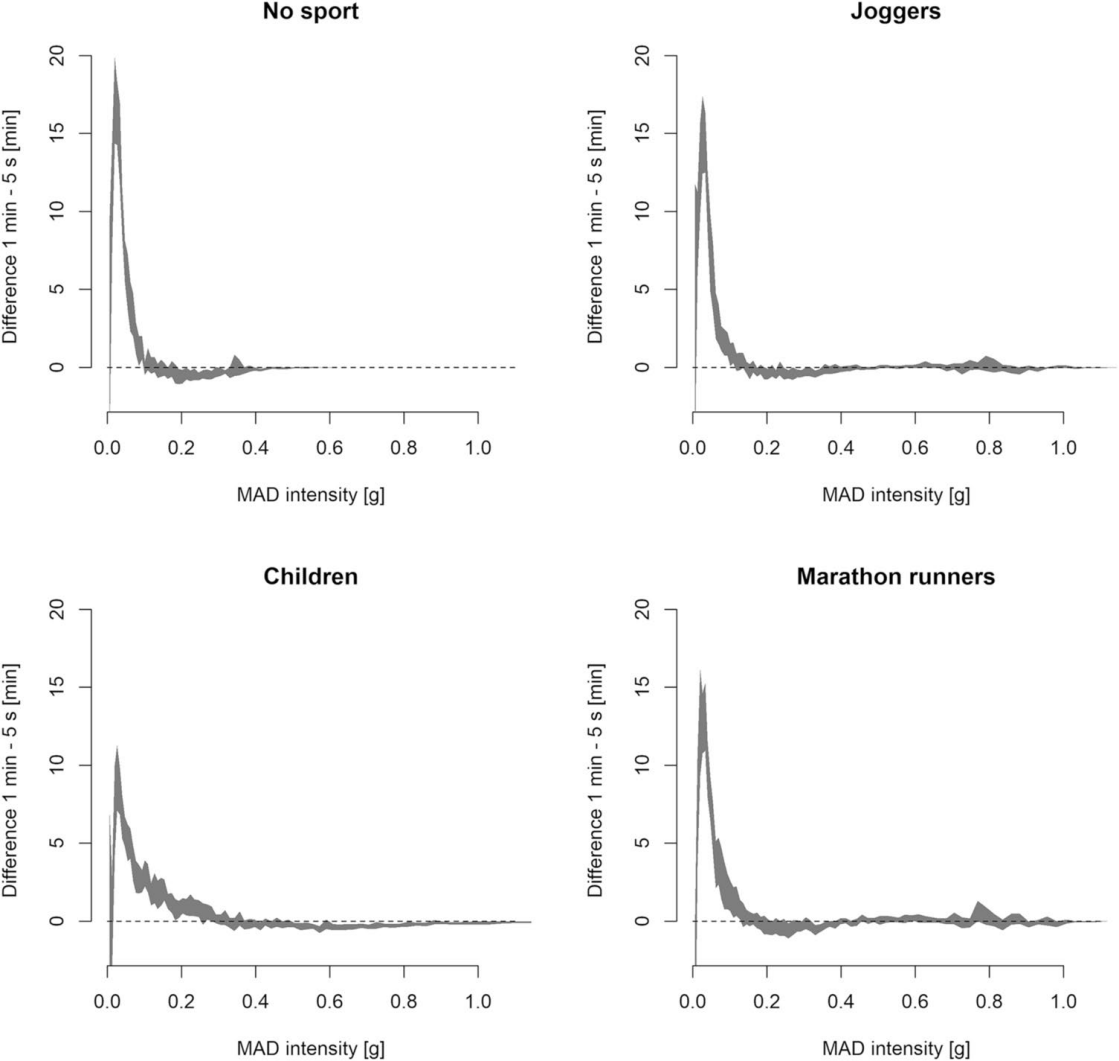
Visualisation of the difference between the 5 s epoch and 1-min epoch activity intensity accumulation histograms. Note that the *Y*-axis has been truncated and the marked difference between the histograms below 0.01 g is not visible. The shaded areas correspond to the 95% confidence interval

Qualitative exploration of the difference in accumulation among the adult groups between the epoch lengths revealed that more minutes were accumulated in the 0.01 to 0.1 g range with the 1 min epoch compared to the 5 s epoch, whereas a slight decrease in accumulation was apparent in the 0.2 to 0.3 g range. The less than 0.01 g range was the most affected with several fewer minutes accumulated in this range with the 1 min epoch compared to the 5 s epoch. The results of all the adult groups were apparently affected in a similar manner by the epoch length. Although a similar overall pattern was apparent among children, the ranges affected differed from the adult groups. That is, the range with additional minutes extended from 0.01 to 0.25 g and the range with fewer minutes from 0.5 to 0.7 g with the less than 0.01 g range behaving similarly to adults in children (Fig. 2).

**Fig. 3:**
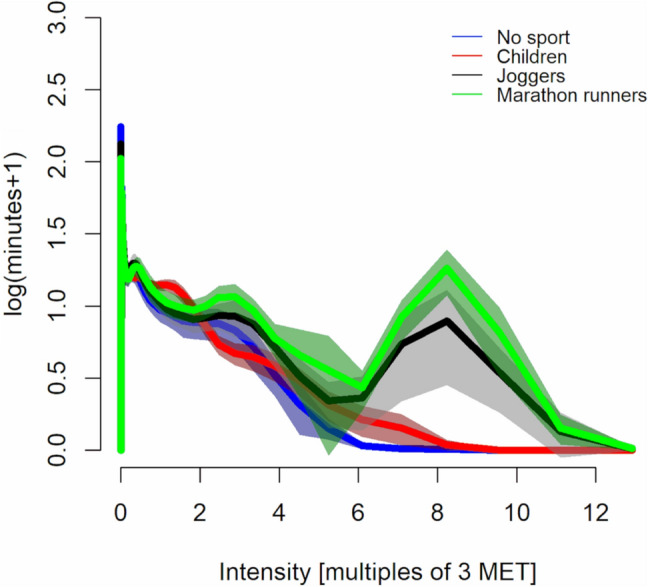
Visualisation of the activity intensity histogram normalised to the intensity corresponding to 3 metabolic equivalents of a task (MET) based on 5 s non-overlapping MAD values. Solid lines are group means, and shaded areas the 95% confidence interval

Figure 3 shows the activity intensity histogram for each group. Exploration of minutes accumulated along the intensity spectrum using intensity normalised to the intensity corresponding to the acceleration of walking at 3 METs with nparLD indicated statistically significant group and intensity bin main effects (ATS 61 to 1120, *df* = 4 to 7, both *p* < 0.001), and a statistically significant group × intensity bin interaction (ATS 23.8, *df* = 19; *p* < 0.001). Kruskal–Wallis comparisons between groups within intensity bins indicated statistically significant between-group differences from 0.74 to 1.58 (all *p* < 0.005), where children accumulated more minutes than any of the adult groups. From 2.47 times the intensity corresponding to the acceleration of walking at 3 METs onwards marathon runners accumulated more minutes than the no sport group (*p* < 0.043). Both the joggers and the marathon runners groups accumulated more minutes than the children and the no sports groups from 7.1 to 11.1 times the acceleration intensity corresponding to walking at 3 METs (*p* < 0.008). Lastly, the marathon runners group accumulated more minutes than all the other groups from 7.1 to 9.6 times the intensity corresponding to the acceleration of walking at 3 METs (*p* < 0.035).

## Discussion

The main findings of the present study were that (1) children accumulated more activity in a band of intensities around the intensity corresponding to the acceleration of walking at 3 METs compared to all three adult groups, and (2) the habitual joggers and marathon runners groups exhibited activity accumulation at intensities corresponding to those observed in running. A conspicuous dose–response relationship was evident with the marathon runners group exhibiting a more pronounced accumulation peak compared to the joggers. Based on these findings children’s free-living activities are lower in intensity than jogging as indicated by the comparison to the adult joggers and runners, and the major bulk falls below the 3 MET intensity. Intensities below 3 METs are likely inadequate to provoke a meaningful improvement in cardiovascular fitness (Armstrong et al. 2011; Collings et al. 2017; Cao et al. 2019), where much more intense activity (intensities corresponding to jogging/running) would be required. That is, although the systematic review by Poitras et al. (2016) indicated a positive association between daily activity (independent of intensity) minutes and physical fitness, they further found that the higher intensity minutes were more indicative of fitness compared to the light activity minutes. Similarly, Armstrong and colleagues (2011) concluded that a structured (and we speculate hence higher intensity) exercise regime may be required to optimise fitness outcomes among children (Armstrong and Barker 2011). The sporadic nature of children’s activity was at least apparently evident in the exploration of the effect of analysis epoch length where the interaction between epoch length and intensity bin counts was stronger in children compared to adults (Fig. 1). That is, the short activity bursts were averaged out in the 1 min epochs and thus masqueraded as lower intensity counts with the 1 min epochs compared to the 5 s epochs. This finding is concordant with the recent and more exhaustive exploration of epoch length-related effects by Aadland et al. (2020).

Troiano and colleagues have reported that children are more physically active than adults based on a US population-representative sample (Troiano et al. 2008). Keeping in mind that our study was more of a proof-of-concept, we would interpret the findings to add to their findings by indicating that most of the activity in children is accumulated at the lighter end (intensities centred around the intensity corresponding to 3 METs) rather than at the higher end of the activity intensity spectrum. The additional knowledge added therefore being the information regarding whether the activity is accumulated closer to the moderate (3 MET) activity threshold or the vigorous (6 MET) threshold even within the intensities that get characterised as moderate with the prevailing four-category analytical approach. These findings lend support to previous research where habitual activity has not been found to be closely associated with cardiorespiratory fitness at low intensities (Collings et al. 2017) but rather higher intensity activities, and participating in sports or exercise seems to be required (Armstrong et al. 2011; Collings et al. 2017). Taking the present findings and past research (Poitras et al. 2016; Aadland et al. 2018b) into consideration, we postulate that high-intensity physical activity rather than the total volume of any physical activity ought to be targeted if attempting to improve cardiorespiratory fitness in children.

Although considering the pattern of activity accumulation along the intensity spectrum is in its infancy (Belavý et al. 2017; Aadland et al. 2018a, b; Rowlands et al. 2018; Migueles et al. 2021), the existing body of literature is encouraging in that it may identify feasible intervention targets not apparent based on more conventional analyses (Belavý et al. 2017; Belavy et al. 2018; Rowlands et al. 2019, 2020; Savikangas et al. 2020). As an example, only relatively short bursts of intense activity are required in high-intensity interval training. Therefore, it is conceivable that the high-intensity end of the activity intensity spectrum could be informative when aiming to maintain or develop cardiovascular fitness. Similarly, bone adaptations require only brief bursts of high-intensity activity, such as jumping, and could also be better represented if high-intensity activities were specifically targeted in numerical processing (Jämsä et al. 2011; Rowlands et al. 2020). These suggestions are in line with what Aadland and colleagues have reported (e.g. Aadland et al. 2018a, b), and contribute to the mounting evidence base, which is starting to suggest that it may be prudent to start testing the hypotheses raised based on these sort of finely graded intensity spectrum explorations formally with randomised controlled trials. That is, the approach of identifying potential intervention targets using such explorations and subsequently getting an efficacious randomised controlled trial outcome with an intervention that modifies the particular specific intensity (as opposed to a broader intensity category such as moderate-to-vigorous physical activity) remains untested in practice to our knowledge.

Some limitations need to be kept in mind when considering the present findings. Firstly, the sample size was rather modest, minimal wear requirement was applied, and convenience samples (rather than e.g. population representative) were recruited. Hence, the results may not be generalisable to the population we sampled from. Secondly, we required only one day of wear to be included in the analysis, when at least three to four days are typically required to ensure a representative sample. This was chosen to maximise the data for analysis in both studies based on the fact that little apparent difference was observed between visualisations of the datasets with all participants included, and only those with three or more valid days (supplementary Fig. 1). Thirdly, we normalised the activity intensities to that corresponding to the acceleration intensity of walking at 3 METs. Whether or not that is reasonable is unclear given there is an ongoing debate as to the use of 4 METs compared to 3 METs for use with (Saint-Maurice et al. 2016; Haapala et al. 2020), however, the normalisation enabled exploring the relative intensity accumulation between children and adults. Visualisation of the intensity spectrum accumulation between the groups with children’s normalised to acceleration intensity of walking at 4 METs provided in supplementary Fig. 2. Fourthly, while we had data from both males and females, the sample size was not sufficient to explore the potential effects of sex. Finally, the CHIPASE dataset was recorded in Finland, and the SAP dataset in Australia. It is plausible that physical activity habits are associated with cultural norms (e.g. Camplain et al. 2020) and the child to adult comparison may be affected by cultural differences. Due to the cross-sectional nature of the study, no cause-effect relationship can be established, and the hypotheses arising from this study would need to be confirmed with prospective trials.

Utilising a finely graded intensity spectrum analysis successfully identified characteristics of behaviour that were expected based on the targeted populations, that is adult runners could be told apart from their non-running peers, and the ones who reported engaging in more running had a more pronounced difference to the non-running peers than the ones who reported engaging in less running. While children seem highly energetic and have been demonstrated to accumulate more total physical activity than adults, children’s physical activity seems to take place at the light-intensity end of the activity spectrum rather than at the high-intensity end. We postulate that this serves as a proof of concept of the intensity spectrum exploration approach and could be extended, for example, into exploring which types of activities might be beneficial for particular health outcomes. For example, children seemed to spend relatively little time in high-intensity activities, and therefore adding targeted higher intensity activities could have the potential to optimise cardiovascular and bone health development.

### Electronic supplementary material

Below is the link to the electronic supplementary material.

Supplementary file1 (TIF 846 KB)

Supplementary file2 (TIF 766 KB)

Supplementary file3 (DOCX 13 KB)

## Data Availability

The datasets during and/or analysed during the current study available from the corresponding author on reasonable request.

## Appendix 1: Technical details of numerical analysis

The approach presented by van Hees et al. (2014) was used in the autocalibration (https://github.com/tjrantal/accelerometer-auto-calibration,commit.f8ca5bb). Briefly, data were pre-processed in 10 s non-overlapping epochs and the epochs with standard deviation less than 0.013 g along all three axes were considered in the calibration. It was possible to create an autocalibration from a recording if at least one epoch with value below – 0.3 g and above 0.3 g was found along each axis (i.e. a minimum of 6 epochs required), and the calibration was calculated using non-linear Levenberg–Marquardt optimisation of the sum:

$$\sum_{e=1}^{N}{\left(\sqrt{{\left({i}_{x}+{s}_{x}{X}_{e}\right)}^{2}+{\left({i}_{y}+{s}_{y}{Y}_{e}\right)}^{2}+{\left({i}_{z}+{s}_{z}{Z}_{e}\right)}^{2}}-1\right)}^{2}$$

where *i* indicates intercept, *s* indicates slope, subscript *x* (*y* or *z*) indicates acceleration axis, *X* (*Y* or *Z*) indicates the mean epoch acceleration along the axis, e indicates the index of a given epoch, and *N* indicates the total number of epochs in consideration. Slopes and intercepts were the parameters that were optimised. Notably, we deviated from the approach suggested by van Hees and colleagues by not implementing weights in the optimisation procedure because our piloting indicated that including weights could result in marked overfitting. All SAP dataset files produced successful autocalibrations. In the CHIPASE dataset some records failed to autocalibrate, and therefore device-specific calibration coefficients were utilised by pooling all calibrations per device, sorting the calibrations in ascending order based on the norm of the slope coefficients, and taking the middle-most calibration as the device calibration.

MAD pre-processing was done in 5 s non-overlapping epochs based on the calibrated resultant acceleration following the procedure presented by Vähä-Ypyä et al. (2015b):

$$\mathrm{MAD }=\frac{\sum_{i=1}^{N}\left|{r}_{i}-\overline{r }\right|}{N}$$

where vertical bars indicate the absolute value, *i* = index of data point, *N* = values in epoch, ${r}_{i}$ = resultant acceleration at sample *i*, and $\overline{r }$ = the mean resultant acceleration of the epoch (Vähä-Ypyä et al. 2015a). Two sets of results were produced from the pre-processed 5 s MAD values. The first set used the 5 s MAD values as is, and the second by calculating minute by minute means. Non-wear was defined independently for both sets as any contiguous epoch of 60 or more minutes with all values below 0.02 g (Savikangas et al. 2020). Day by day histograms expressed as minutes per day were produced from both sets of results by dividing the MAD values into a hundred bin logarithms day by day. The histogram bins ranged logarithmically equidistantly from 0 to 2. In practice, bin thresholds were decided by the following procedure: exponential values were calculated by raising e (~ 2.72) to powers from 0 to 2 with an increment of 2/100. Next, one was taken off from all exponential values. The remaining values were normalised to the maximum of the remaining values, and finally multiplied by 2.

## Abbreviations

ATS: Anova-type statistic
CHIPASE: Children’s physical activity spectrum study
MAD: Mean amplitude deviation
MET: Metabolic equivalent of a task
nparLD: Rank-based non-parametric methods for analysing longitudinal data in factorial experiments
SAP: Spine activity study
SD: Standard deviation
