# Supplementary material for: Physical activity accumulation along the intensity spectrum differs between children and adults
Source: Eur J Appl Physiol. 2021 Jun 5;121(9):2563–71. doi: 10.1007/s00421-021-04731-3 (PMC8357734; doi:10.1007/s00421-021-04731-3)
Supplement: Supplementary file 3 — Supplementary file3 (DOCX 13 KB) [file 421_2021_4731_MOESM3_ESM.docx]

**SUPPLEMENTAL FIGURE LEGENDS**

**Figure S1. Visualisation of the activity intensity histograms based on one minute non-overlapping activity intensity processing (black line and darker shading), and 5 second non-overlapping activity intensity mean amplitude deviation (MAD) processing (grey line and lighter shading). Only participants with at least 3 days wear included. Mean values plotted with solid lines, and the shaded areas correspond to the 95% confidence interval.**

**Figure S2. Visualisation of the activity intensity histogram normalised to the intensity corresponding to 3 metabolic equivalents of a task (MET) for adults, and 4 METs for children based on 5 second non-overlapping MAD values. Solid lines are group means, and shaded areas the 95% confidence interval**
